# Oral Health Status and Oral Health-Related Behaviours of Hong Kong Students with Vision Impairment

**DOI:** 10.3390/healthcare12030391

**Published:** 2024-02-02

**Authors:** Jessica Ka Yi Lee, Agatha Wing Tung Yuen, Karen Pui Yan Leung, Joyce Tin Wing Li, Seon Yeong Bae, Yi Yung Chan, Ching Kiu Ip, Sik Hong Lau, Yin Ngai Lau, Hei Yuet Lo, Shuk Kwan Tang, Duangporn Duangthip

**Affiliations:** 1 Faculty of Dentistry, The University of Hong Kong, Hong Kong, China; 2 College of Dentistry, The Ohio State University, Columbus, OH 43210, USA

**Keywords:** dental caries, oral hygiene, oral health, vision disorders, epidemiology, community dentistry

## Abstract

This cross-sectional epidemiological study aimed to describe dental caries and oral hygiene conditions among visually impaired students in Hong Kong. Students aged 6–21 years from two specialised schools for those with vision impairment were invited. Information on sociodemographic background and oral health-related behaviours was collected through an online parent-reported questionnaire. Dental caries and oral hygiene were assessed using the Decayed, Missing and Filled Teeth (DMFT) index and the Visible Plaque Index (VPI), respectively. Chi-square, Mann–Whitney U and Kruskal–Wallis H tests were conducted to analyse the association between students’ background and oral health status. A total of 73 participants were recruited, of whom 57.5% were male. Their mean (SD) age was 12.9 (4.7) years. Their mean DMFT score (SD) was 1.0 (1.8), and 43.8% had caries experience. The mean VPI (SD) was 0.76 (0.30). Their caries experience was significantly associated with their snacking habits (*p* = 0.013). Male participants had poorer oral hygiene than females (*p* = 0.048). In summary, dental caries is prevalent among visually impaired students in Hong Kong and their oral hygiene condition is unsatisfactory. Caries experience is significantly associated with snacking frequency, whereas oral hygiene is associated with gender. More specially designed preventive oral health measures should be provided for visually impaired students and their caretakers.

## 1. Introduction

Vision impairment, as defined by the World Health Organization (WHO), is one of the most common disabilities worldwide, affecting 596 million people, which includes 1.4 million children suffering from blindness and 22.6 million children suffering from moderate-to-severe sight loss [1]. Blindness is defined as visual acuity worse than 3/60, and moderate-to-severe vision impairment is defined as worse than 6/18 but equal to or better than 3/60 [2]. Vision loss hampers children’s academic performance [3], development of social skills and general well-being [4].

Systematic reviews [5] and epidemiological studies in Northeast China [6], Saudi Arabia [7] and India [8,9] have shown that individuals with vision impairment had a higher prevalence of dental caries as well as more gingival inflammation and dental trauma than their sighted counterparts. Visually impaired children may face difficulty in performing proper home care for oral health due to their inability to visualise dental plaque and lack of hand–eye coordination [10]. In addition, the oral health care of the disabled may often be overlooked [11]. Dental treatment needs for populations with special care needs are often greatly underestimated by their caregivers [12]. Despite sufficient financial support, 20% of children with special care needs had unmet dental treatment needs [13], possibly due to the obstacles that they face when seeking dental care (e.g., dental phobia, treatment complications posed by their medical conditions, lack of information and difficulty finding a dentist who is willing to provide care) [13]. Similarly, visually impaired individuals also had poorer access to dental care due to their physical, social and information barriers [14,15].

In Hong Kong, limited dental services are provided to children requiring special care. There are dental clinics set up for intellectually impaired patients [16], yet there is no equivalent care for people with reduced vision. Children in primary school (aged 6–12 years) are covered under the School Dental Care Services (SDCS), in which dental examination as well as preventive and restorative dental treatments, such as fluoride application, scaling, dental filling, etc., are provided annually [17]. Although visually impaired students are eligible to join this program, they may not be able to receive modified oral hygiene instructions due to limited time and resources in such a setting [18]. In addition, the scheme does not cover preschool or secondary school students.

The latest Special Topics Report issued by the Hong Kong Census and Statistics Department in 2021 stated that approximately 47,600 people were suffering from various degrees of visual impairment. Among them, 600 were younger than 15 years old and more than half (61.7%) of the entire population with visual impairment had multiple disabilities [19]. Despite the prevalence of visual impairment, no updated published study about the oral health status of this group in Hong Kong is available (searched in PUBMED on 1 October 2023). Therefore, a survey about the oral health status of visually impaired students is necessary for policymakers and dental practitioners to plan and provide individualised oral health care for the visually impaired in Hong Kong.

This study aimed to describe the dental caries and oral hygiene status of visually impaired students in Hong Kong and investigate the risk factors associated with oral health status. The secondary objective was to describe this population’s perceived barriers to dental care services.

## 2. Materials and Methods

The Institutional Review Board of the University of Hong Kong/Hospital Authority Hong Kong West Cluster (IRB UW 23-480) approved the current study. Written consent was obtained from the participants’ parents/guardians prior to the day of service. This study was conducted in full accordance with ethical principles. This cross-sectional oral health survey was conducted in March 2023.

### 2.1. Sample Selection

In Hong Kong, there are two schools dedicated to students with vision impairment: Ebenezer School and Ebenezer New Hope School. The screening process involved all students from both schools (*n* = 126). Students who had very severe intellectual disabilities or complex medical diseases that hindered their ability to cooperate with dental examination were excluded. Subsequently, the remaining students (*n* = 107) who met the inclusion criteria of being over five years old and having vision impairment were invited to participate.

### 2.2. Questionnaire

Before the survey was conducted, a consent form and a self-completed questionnaire were distributed to each of the invited schoolchildren’s parents. The questionnaire consisted of four parts: (i) child’s background: sex, age and vision impairment severity; (ii) socioeconomic background: parents’ education level and family’s monthly income; (iii) oral health-related habits: toothbrushing habits, interdental cleaning habits, oral hygiene instruction experience and snacking habits; and (iv) access to oral health practitioners and information: frequency, difficulty accessing and sources of oral health information.

### 2.3. Clinical Examination

This study was conducted in an outreach setting where an experienced dental epidemiologist (DD) trained and supervised three examiners. Each examiner’s first two cases were calibrated with the supervisor (DD). Afterward, all the cases in the first half of the survey were checked by DD to ensure accuracy. The visually impaired students were positioned supine on portable dental chairs with manually adjustable back height and inclination at the school. The clinical examinations were conducted using a straight probe, a ball-ended WHO Community Periodontal Index probe and an LED light handle with a mirror attached (MirrorLite, Kudos Crowns Limited, Hong Kong SAR, China). 

Caries experience was recorded using the decayed, missing and filled primary teeth (dmft) and permanent teeth (DMFT) indices, following the WHO’s diagnostic criteria [20]. A tooth was considered ‘decayed’ (dt/DT) if there was a remarkable caries cavity. A tooth was considered ‘missing’ (mt/MT) if it was extracted due to caries. A tooth was considered ‘filled’ (ft/ FT) if there was a permanent filling without caries. DMFT scores were recorded for all participants, whereas dmft scores were only noted for those under the age of 12. The participants’ oral hygiene status was denoted using the Visible Plaque Index (VPI) [21]. The buccal and lingual surfaces of index teeth—upper right first molar (16), upper right central incisor (11), upper left first molar (26), lower left first molar (36), lower right central incisor (41) and lower right first molar (46)—were examined for plaque, and the presence of plaque was noted as ‘1′ (regardless of the thickness), whereas the absence of plaque was noted as ‘0′. The VPI score was then calculated as the ratio of the number of surfaces with plaque to the total number of surfaces examined.

Following a student’s oral examination, their oral health report, which included a summary of preliminary dental findings and recommendations for each student, was sent to their parents. Dental treatments were provided based on the individual’s needs and within the project’s constraints. Topical 5% sodium fluoride varnish (Duraphat, Pharbil Waltrop GmbH, Waltrop, Germany) and 38% silver diamine fluoride solution (Saforide, Toyo Seiyaku Kasei Co., Ltd., Osaka, Japan) were applied as necessary for primary and secondary caries prevention, respectively. A tailored oral health instruction using auditory and tactile learning strategies was provided after the examination and treatment. This involved clear verbal explanations of oral care steps, physical demonstrations and hands-on guidance for brushing and flossing practices.

### 2.4. Statistical Analysis

Data analysis was conducted using SPSS 24.0 for Windows (SPSS Inc., Chicago, IL, USA). Univariate analysis was adopted to analyse the independent variables’ effects on caries prevalence and oral hygiene status. Chi-square tests were conducted to analyse caries experience (yes/no) according to the students’ demographic characteristics (age, sex, severity of vision impairment, parents’ education levels and family income) and oral health habits (frequency of toothbrushing and interdental cleaning, oral hygiene instruction experience, frequency of snacking and dental visit experience). For the assessment of oral hygiene status, the Shapiro–Wilk test was used to test the normality of the VPI data. Because the results indicated that the VPI data were non-parametric, the Mann–Whitney U test and Kruskal–Wallis H tests were conducted to analyse the association between oral hygiene status (VPI) and the variables described above. The level of statistical significance was set at 0.05.

## 3. Results

**Figure 1:**
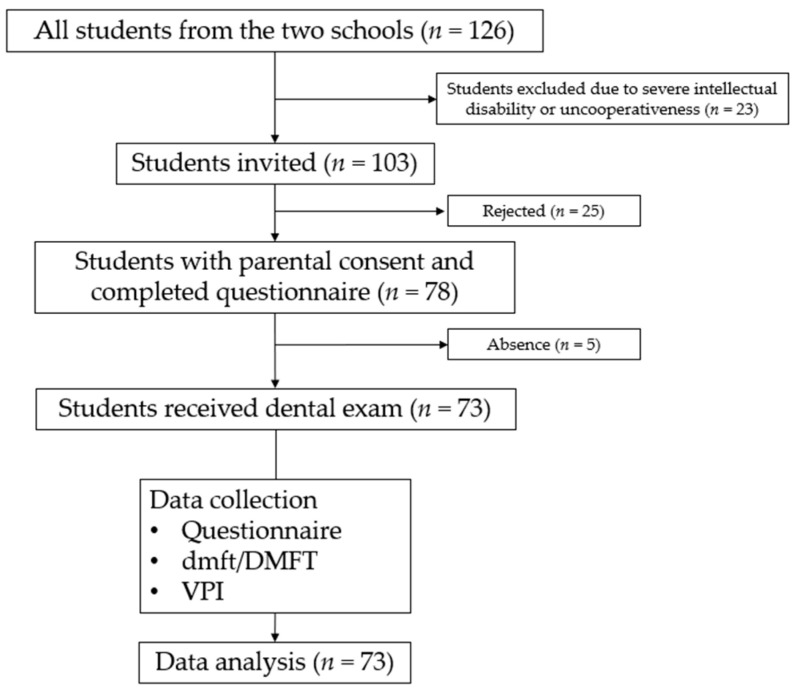
Participant recruitment flowchart.

Among the 103 students invited, 78 students (75.7% response rate, 78/103) completed the questionnaire and consented to examination and fluoride treatment. On the day of the examination, five students were absent. Thus, 73 students (70.9% participation rate, 73/103) joined the project. The flowchart of participants’ recruitment is shown in Figure 1.

### 3.1. Sociodemographic Characteristics

Of the study participants, 31 were female (42.5%) and 42 male (57.5%). The age range was 6–21 years, with a mean age (SD) of 12.9 (4.7) years. The numbers of participants who were 6–11 years old, 12–14 years old and 15–21 years old were 32 (43.8%), 14 (19.2%) and 27 (37.0%), respectively. Regarding the severity of vision impairment, one-third (31.5%) of the participants were unable to see at all, one-third (34.2%) required special visual aids and the rest (34.2%) did not need special visual aids. Table 1 summarises the visually impaired students’ backgrounds.

### 3.2. Oral Health Status

#### 3.2.1. Caries Status

- Overall, nearly half (43.8%, 32/73) of the participants had caries experiences (dmft or DMFT > 0). Their mean DMFT score (SD) was 1.0 (1.8) (Table 2).
- Caries experience in primary teeth: For children aged 6–11, their mean dmft score (SD) was 2.5 (4.2). The mean (SD) number of untreated decayed teeth (dt) was 2.4 (4.2), constituting more than 95% of the dmft score. The mean number of filled or missing teeth was small (ft = 0.1; mt = 0.04). A positively skewed distribution of the dmft score was found, with the skewness being 2.0.
- Caries experience in permanent teeth: The overall mean (SD) DMFT score was 1.0 (1.8). Altogether, DT (0.8) constituted 80% of the DMFT score. The mean number of FT and MT across all age groups was small (FT = 0.2; MT ≤ 0.1), except in the 15–21 age group, in which a larger proportion of FT was observed (0.5). A positively skewed distribution of the DMFT score was found, with the skewness being 2.3.

#### 3.2.2. Oral Hygiene Status

For participants aged 6–11, 12–14 and 15 or above, the mean VPI was 0.69, 0.90 and 0.78, respectively (Table 2). The overall mean for VPI across all three age groups was 0.76, with 1 as the total score.

### 3.3. Oral Health Behaviours

More than half of the participants (57.5%) brushed their teeth independently. Almost all of them (90.4%) brushed twice a day, and 20.5% of them performed interdental cleaning regularly. Most (82.2%) had a daily snacking habit, and one-third of them (31.5%) snacked twice or more daily. Chips and biscuits (39.7%) were the most popular snacks, followed by candies and chocolates (26.0%), dairy products (21.9%) and sweetened drinks (11.0%).

A large proportion (87.6%) of participants had dental visit experience. Three-quarters (74.0%) went to the SDCS, and the rest visited dentists at private clinics (11%), government dental clinics (9.6%) and charitable organisations (1.4%). However, almost half of the parents or caregivers (43.8%, 32/73) reported facing difficulties when they sought dental care for their children. Of those 32 parents or caregivers, 20 (62.5%) expressed that the child’s uncooperative behaviour was the main barrier. Almost half of these 32 parents (46.9%) found it difficult to find a suitable dentist to manage their child with special needs and were concerned about the cost of dental treatment.

### 3.4. Univariate Analysis

Caries prevalence was significantly associated with snacking frequency (Table 3). Students who snacked twice or more per day had a higher prevalence of caries than those who snacked once or not at all daily (*p* = 0.013). The other independent factors were not statistically associated with caries experience (*p* > 0.05).

Poor oral hygiene was associated with gender (Table 4). In the Mann–Whitney U test, a significantly higher VPI score was observed among the male students than among the female students (*p* = 0.048). The other factors were not statistically associated with VPI.

## 4. Discussion

Previous studies have shown that visually impaired children have higher caries prevalence, poorer oral hygiene and higher dental trauma experience than their peers with normal vision [5,6,7,8,9,15]. Similarly to the results of the current study, visually impaired school students had a poorer oral health status than their non-impaired counterparts participating in the territory-wide Oral Health Survey (OHS) in Hong Kong [22]. The participants aged 12 to 14 in this study had a higher mean DMFT score (1.1) and higher caries prevalence (35.7%) than their sighted peers in the OHS (mean DMFT: 0.4, prevalence: 22.6%). These participants also displayed a higher mean VPI in mixed (0.69 vs. 0.22) and permanent dentition (0.90 vs. 0.27) than their sighted counterparts of similar age in the aforementioned survey. This study’s results also coincided with a systematic review and meta-analysis by Costa Silva-Freire et al. [5], which showed that visually impaired children had poorer oral health. These data suggest that the inability to see hinders oral health because dental plaque cannot be effectively removed during routine cleaning at home.

In the present study, the frequency of daily snacking was found to be significant for caries prevalence (*p* = 0.013). Participants who snacked twice or more per day had a higher caries prevalence. This association is also corroborated by other studies [23,24] and aligns with the current understanding that when the demineralisation rate caused by bacterial acid outweighs the remineralisation rate, caries results [25]. Although parents may use snacks as a reward for their schoolchildren [26], students with vision impairment might not be able to see and select the type of food and snack that they consume by themselves [27]; therefore, parents and caretakers of visually impaired students should be educated on the correlation of daily snacking with dental caries.

Regarding oral hygiene status, female participants had a significantly lower VPI than their male counterparts. Other studies [28,29,30,31,32] with subjects whose vision was not impaired have also shown that females had better oral hygiene practice and lower gingival index than males. This finding may be attributed to the fact that females are more likely to take notice of their oral hygiene and therefore are more motivated to perform proper oral hygiene practices. As a result, future preventive programs may provide more intensive oral hygiene training for male participants.

Contrary to the belief that regular dental visits and receiving oral preventive measures enhance oral health [33], a higher caries rate was observed among participants who had visited a dentist or received oral hygiene instruction. This implies that dental visits had been sought because these students had already encountered oral health problems. In this study, the dental visit rate was high, possibly revealing a high treatment need for visually impaired students. Despite the high visit rate, almost half of the parents of visually impaired students had faced difficulty in accessing suitable dental care for their child. The most reported hurdle was the child’s uncooperative behaviours, followed by cost and difficulty in finding suitable dentists. Also, a comprehensive list of dentists who can manage children with special care is difficult to find. It is particularly crucial for students in secondary school to find appropriate dentists because they are no longer included in the SDCS scheme. In general, the higher prevalence of untreated caries and poorer oral hygiene observed in visually impaired students may have occurred due to poor access to dental services.

Visually impaired individuals may face great fear when visiting a dentist because they cannot visualise the surrounding environment and may be more sensitive to noise and other environmental stimuli. A recent study in India [34] showed that almost all (90%) children with vision impairment had dental anxiety before a dental visit and more than half of them had severe dental anxiety. Also, oral hygiene instruction for sighted individuals generally includes the use of disclosing tablets or other visual aids to indicate dental plaque, as well as the use of a tooth model to illustrate proper brushing technique. Because these measures are not suitable for the visually impaired, modified oral hygiene techniques should be taught to this population, focusing on audio and tactile stimulation [18,35]. Given that “special care dentistry” is not available as a specialty in Hong Kong, the major dental care providers for visually impaired individuals would be general dentists. Therefore, general practitioners need to adopt small changes in their practices to deliver better oral health care for these patients [36]. To improve the oral health of this vulnerable group, dental professionals should utilise adaptive technologies and offer education in alternative formats for visually impaired populations. Oral health policies should also advocate for inclusive dental services, professional training requirements and increased research funding for innovative solutions. This collaborative effort may break barriers and enhance oral health outcomes for visually impaired students with high caries risk and poor oral hygiene.

Although this study had a high response rate from the two specialised schools for visually impaired students in Hong Kong, the sample size was limited. The majority of students with mild vision impairment attend other schools with a regular curriculum, and those with complex medical problems may attend other specialised healthcare institutions. In addition, students in these two schools with severe intellectual disability were unable to undergo a dental examination. As a result, the findings may not be applicable to the entire population of visually impaired students in Hong Kong. Furthermore, because the information about the students’ sociodemographic characteristics and oral health behaviour was collected via a parent-reported questionnaire at one point, the proxy response, recall biases and other confounding factors may have influenced the results, reducing its accuracy. Also, due to the time limitation and dependence on children’s cooperation, test–retest and interrater calibration were not performed in this study. This may reduce the reliability of the study. However, a specialist in dental public health supervised all the examinations to ensure the results’ accuracy and consistency. Further cohort studies with a larger sample size should be conducted to investigate the relationship between the oral health status of students with vision impairment and potential factors in Hong Kong. Nevertheless, the present study provided novel data regarding the caries prevalence, oral hygiene status and barriers to dental visits of visually impaired students in Hong Kong.

## 5. Conclusions

In summary, dental caries is prevalent among visually impaired students in Hong Kong. Their oral hygiene is unsatisfactory. Caries experience is significantly associated with snacking frequency whereas poor oral hygiene is associated with gender. Children’s uncooperative behaviour is reported by parents as the most common barrier to visually impaired students’ access to dental care. It is recommended that more oral education information focusing on snacking and dental caries should be provided to visually impaired students and their caregivers to raise their awareness of caries prevention. Also, extended oral hygiene training could be provided to male students to enhance their skills and motivation in home care practice. Furthermore, policymakers should consider providing additional training for dental practitioners, and subsidisation for oral healthcare for the visually impaired in Hong Kong.

## Figures and Tables

**Table 1:** The participants’ sociodemographic characteristics.

Sociodemographic Characteristics		*N*	Frequency
Age (years)	6–11	32	43.8%
	12–14	14	19.2%
	15–21	27	37.0%
Sex	Female	31	42.5%
	Male	42	57.5%
Severity of vision impairment	Unable to see at all	23	31.5%
	Requires special visual aid	25	34.2%
	Does not require special visual aid	25	34.2%
Family monthly income (HKD)	≤HKD 16,000	15	20.5%
	HKD 16,001–30,000	21	28.8%
	HKD 30,001–50,000	22	30.1%
	>HKD 50,000	15	20.5%

**Table 2:** The participants’ dental caries and oral hygiene status in permanent teeth.

Age (Years)	*N*	D Mean (SD)	M Mean (SD)	F Mean (SD)	DMFT Mean (SD)	Caries Prevalence (%)	VPI Mean (SD)
6–11	32	0.7 (1.6)	0.0 (0.0)	0.07 (0.4)	0.8 (1.6)	31.1	0.69 (0.33)
12–14	14	1.0 (1.8)	0.0 (0.0)	0.07 (0.3)	1.1 (1.8)	35.7	0.90 (0.16)
15–21	27	0.7 (1.4)	0.0 (0.0)	0.5 (1.1)	1.2 (1.9)	40.7	0.78 (0.29)
Overall	73	0.8 (1.6)	0.0 (0.0)	0.2 (0.8)	1.0 (1.8)	35.5	0.76 (0.30)

Abbreviations: D = decay without restoration in permanent teeth, M = missing tooth due to caries in permanent teeth, F = filled tooth in permanent teeth; DMFT = the number of decayed, missing and filled permanent teeth; VPI = Visible Plaque Index.

**Table 3:** Association between caries prevalence and potential factors.

Independent Factors		*N*	Caries Prevalence	*p*-Value *
Age (years)	6–11	32	50.0%	0.614
	12–14	14	35.7%	
	15–21	27	40.7%	
Sex	Female	31	51.6%	0.250
	Male	42	38.1%	
Severity of vision impairment	Unable to see at all	23	43.5%	0.522
	Requires special visual aid	25	36.0%	
	Does not require special visual aid	25	52.0%	
Father’s education level	Primary or below	5	20.0%	0.531
	Junior secondary	16	56.3%	
	Senior secondary	26	42.3%	
	Post-secondary	26	42.3%	
Mother’s education level	Primary or below	7	28.6%	0.590
	Junior secondary	12	58.3%	
	Senior secondary	31	45.2%	
	Post-secondary	23	39.1%	
Family monthly income (HKD)	≤HKD 16,000	15	60.0%	0.503
	HKD 16,001–30,000	21	42.9%	
	HKD 30,001–50,000	22	40.9%	
	>HKD 50,000	15	33.3%	
Received oral hygiene instruction previously	Yes	51	51.0%	0.061
	No	22	27.3%	
Dental check-up experience	Yes	64	46.9%	0.163
	No	9	22.2%	
Frequency of daily snacking	0–1	50	34.0%	0.013
	≥2	23	65.2%	
Brushing habit	Independent brushing	42	47.6%	0.638
	Parent-supervised brushing	7	42.9%	
	Parent-assisted brushing	24	37.5%	
Frequency of daily brushing	0–1	7	14.3%	0.097
	≥2	66	47.0%	
Frequency of daily interdental cleaning	0	58	44.8%	0.737
	≥1	15	40.0%	

* Chi-squared test.

**Table 4:** Association between Visible Plaque Index (VPI) and potential factors.

Independent Factors		Median	*p*-Value
Age (years)	6–11	0.75	0.070 ^b^
	12–14	1.00	
	15–21	0.92	
Sex	Female	0.75	0.048 ^a^
	Male	1.00	
Severity of vision impairment	Unable to see at all	0.83	0.586 ^b^
	Requires special visual aid	0.75	
	Does not require special visual aid	1.00	
Father’s education level	Primary or below	1.00	0.287 ^b^
	Junior secondary	0.67	
	Senior secondary	0.83	
	Post-secondary	0.10	
Mother’s education level	Primary or below	0.75	0.593 ^b^
	Junior secondary	0.92	
	Senior secondary	1.00	
	Post-secondary	0.75	
Family monthly income (HKD)	<HKD 16,000	1.00	0.560 ^b^
	HKD 16,001–30,000	0.75	
	HKD 30,001–50,000	0.83	
	>HKD 50,000	1.00	
Received oral hygiene instruction previously	Yes	0.75	0.120 ^a^
	No	1.00	
Dental check-up experience	Yes	0.88	0.103 ^a^
	No	0.58	
Frequency of daily snacking	0–1	1.00	0.178 ^a^
	≥2	0.83	
Brushing habit	Independent brushing	0.79	0.216 ^b^
	Parent-supervised brushing	0.75	
	Parent-assisted brushing	1.00	
Frequency of daily brushing	0–1	1.00	0.628 ^a^
	≥2	0.83	
Frequency of daily interdental cleaning	0	1.00	0.103 ^a^
	≥1	0.67	

^a^ Mann–Whitney U test, ^b^ Kruskal–Wallis H test.

## Data Availability

The data presented in this study are available on request from the corresponding author.
